# Profiling of circulating tumor DNA in plasma of non‐small cell lung cancer patients, monitoring of epidermal growth factor receptor p.T790M mutated allelic fraction using beads, emulsion, amplification, and magnetics companion assay and evaluation in future application in mimicking circulating tumor cells

**DOI:** 10.1002/cam4.2244

**Published:** 2019-05-21

**Authors:** Jessica Garcia, Anne‐Sophie Wozny, Florence Geiguer, Aurélia Delherme, David Barthelemy, Patrick Merle, Claire Tissot, Frederick S. Jones, Chassidy Johnson, Xiaobin Xing, Zhenyu Xu, Daniel L. Edelstein, Marie Brevet, Pierre‐Jean Souquet, Claire Rodriguez‐Lafrasse, Léa Payen, Sébastien Couraud

**Affiliations:** ^1^ Laboratoire de Biochimie et Biologie Moléculaire, Groupe Hospitalier Sud Hospices Civils de Lyon Lyon France; ^2^ Cancer Research Center of Lyon, INSERM U1052, CNRS UMR5286 Claude Bernard University, University of Lyon Lyon France; ^3^ CIRculating CANcer (CIRCAN) program Hospices Civils de Lyon Cancer institute Lyon France; ^4^ Laboratoire Commun de Recherche Hospices Civils de Lyon – BioMérieux, Centre Hospitalier Lyon Sud Hospices Civils de Lyon Lyon France; ^5^ Service de Pneumologie et oncologie thoracique CHU G Montpied Clermont‐Ferrand France; ^6^ Service de Pneumologie et Cancérologie Thoracique CHU Saint Etienne Saint‐Priest‐en‐Jarez France; ^7^ Medical Scientific Affairs Sysmex Inostics, GmBH Hamburg Germany; ^8^ Biolidics Limited Singapore Singapore; ^9^ SOPHiA GENETICS SA, Headquarters Saint Sulpice Switzerland; ^10^ Institut de pathologie multisites des HCL‐Site Est, Hospices Civils de Lyon Lyon France; ^11^ Service de Pneumologie aigue spécialisée et cancérologie thoracique Groupement hospitalier sud, Institut de Cancérologie des Hospices Civils de Lyon Lyon France; ^12^ UMR CNRS 5822/IN2P3, IPNL, PRISME, Laboratoire de Radiobiologie Cellulaire et Moléculaire, Faculté de Médecine Lyon‐Sud Université Lyon 1 Lyon France; ^13^ EMR 3738 Ciblage Thérapeutique en Oncologie, Faculté de médecine Lyon Sud Université Lyon 1, Université de Lyon Lyon France

**Keywords:** circulating‐free DNA, digital PCR, liquid biopsy, lung cancer, NGS

## Abstract

Cell‐free plasma DNA (cfDNA) and mimicking circulating tumor cells (mCTCs) have demonstrated tremendous potential for molecular diagnosis of cancer and have been rapidly implemented in specific settings. However, widespread clinical adoption still faces some obstacles. The purpose was to compare the performance of a BEAMing (beads, emulsion, amplification, and magnetics) assay (OncoBEAM™‐epidermal growth factor receptor [*EGFR*] [Sysmex Inostics]) and a next‐generation sequencing assay (NGS; 56G Oncology panel kit, Swift Bioscience) to detect the p.T790M *EGFR* mutation in cfDNA of non‐small cell lung cancer (NSCLC) patients. CfDNA samples (n = 183) were collected within our hospital from patients having a known *EGFR* sensitizing mutation, and presenting disease progression while under first‐line therapy. *EGFR* mutations were detected using NGS in 42.1% of samples during progression in cfDNA. Testing using the OncoBEAM™‐*EGFR* assay enabled detection of the p.T790M *EGFR* mutation in 40/183 NSCLC patients (21.8%) *versus* 20/183 (10.9%), using the NGS assay. Samples that were only positive with the OncoBEAM™‐*EGFR* assay had lower mutant allelic fractions (Mean = 0.1304%; SD ± 0.1463%). In addition, we investigated the detection of p.T790M in mCTCs using H1975 cells. These cells spiked into whole blood were enriched using the ClearCellFX1 microfluidic device*.* Using the OncoBEAM™‐*EGFR* assay, p.T790M was detected in as few as 1.33 tumoral cells/mL. Overall, these findings highlight the value of using the OncoBEAM™‐*EGFR* to optimize detection of the p.T790M mutation, as well as the complementary clinical value that each of the mutation detection assay offers: NGS enabled the detection of mutations in other oncogenes that may be relevant to secondary resistance mechanisms, whereas the OncoBEAM™‐*EGFR* assay achieved higher sensitivity for detection of clinically actionable mutations.

## 1 INTRODUCTION

Knowledge of the molecular complexity of cancers and the role of oncogenic drivers such as mutations in genes encoding the Epidermal Growth Factor Receptor (*EGFR*) has flourished in the era of targeted therapies.^1^, ^2^ Molecular profiling of a cancer patient's tumor to reveal targetable alterations is an important first step in the personalization of cancer treatment plans. Usually, these molecular mutational analyses are performed on Formalin‐Fixed Paraffin‐embedded (FFPE) tissue biopsies or cytological samples at diagnosis or upon recurrence.^3^ Mutational analysis of FFPE tumor samples, however, poses several limitations and challenges, including the invasive procedures required to obtain them, as well as the risk of obtaining false‐negative results due to tumor spatial heterogeneity or low tumor cellularity.^4^, ^5^ Biopsies of advanced stage Non‐Small Cell Lung Cancers (NSCLC) require invasive exams in fragile patients and “liquid biopsies” have generated a considerable enthusiasm in this particular setting. Reassessing molecular alterations at progression is crucial in some particular settings of NSCLC. Indeed, the use of cell‐free DNA (cfDNA, including DNA from normal cells and cancer cells) for the detection of sensitizing and/or resistant somatic alterations in oncodrivers was integrated to the EMA approval. The use of circulating tumor cells (CTCs), although not yet routinely used, has led to very promising complementary results in the context of molecular and phenotypic characterization, prognosis and predictive significance in NSCLC (DNAseq, RNAseq, miRNA and protein). CTCs are cells from the tumor, whole and living, circulating in the whole blood.

Temporal heterogeneity is also a major limitation, particularly if a targetable alteration such as the p.T790M *EGFR* mutation emerges in response to first‐line tyrosine kinase inhibitor (TKI) therapy. Particularly, challenging in NSCLC is the restrieval of adequate tissue biopsy specimens for molecular analysis at diagnosis, which is not possible in approximately 10‐15% of advanced or metastatic NSCLC. Currently, few rebiopsies are performed in advanced NSCLC due to the invasiveness of the procedure, combined with the reluctance of patients with a poor performance status to be rebiopsied. However, it is not possible to properly target treatments in cases of relapse without knowing the nature of the disease during progression. In an analysis of complication rates of biopsies obtained in 57 clinical trials, the complication rate for intrathoracic biopsies was 17.1%, or 36 of 211 biopsies.^6^, ^7^Another major issue is the highly variable and extended time it takes for tissue biopsy results to return to the clinic versus the urgent need to begin treatment in patients with advanced disease. The time lapse between a physician's request and receipt of molecular diagnostic test results is highly variable across the world. In one report the average time to clinical reporting was 27 days with a range of 14 to 77 days.^8^ Altogether, these limitations prompt the need for accurate and sensitive mutation detection technologies that do not rely on invasive tumor tissue sampling. Sensitive detection technologies capable of accurately detecting mutations in plasma samples obtained from blood provide a minimally invasive alternative to tissue‐based methods.^9^, ^10^, ^11^ This clinical need has prompted the development and regulatory approval in the United States and Europe of plasma *EGFR* companion diagnostic tests based on either real‐time or conventional polymerase chain reaction (PCR).^12^ Conventional and real‐time PCR carries out one reaction per single sample and provides one signal. Thus, with conventional PCR if a rare mutant molecule is present and provides a weak signal, it may not be detected in the sample, because the signal is lost among the abundant nontumor signal, as well as signal from tumor DNA unrelated to the mutation. To this end, highly sensitive and selective technologies have been developed to overcome the inherent challenge of obtaining a strong signal from the very low fraction of tumor‐derived DNA in comparison to the wild‐type (WT) fraction.^11^ In this regard, BEAMing (beads, emulsion, amplification, and magnetics), a technology based on digital PCR, which entails partitioning the PCR process into many individual reactions in order to provide higher resolution for the less frequently encountered DNA sequences (ie mutated DNA), appears to be a highly promising technique. It can identify mutations at a mutant allele fraction (MAF) of 0.02% and has previously been demonstrated to be among the most sensitive methods to detect mutations from circulating tumor DNA (ctDNA).^11^, ^13^ In addition, next‐generation sequencing (NGS) technologies, including the 56G Oncology panel (NGS‐56G) are now demonstrating sensitivity levels as low as 0.5% MAF in clinical use.^11^ One remaining hurdle is the identification of somatic alterations in patients whose tumor biology does not provide sufficient levels of ctDNA material to allow detection. The proportion of patients with detectable ctDNA varies by indication,^14^ stage of disease,^15^ tumor burden, tumor location and other biological characteristics that may be unrelated to the tumor.^16^, ^17^ In *EGFR*‐mutated NSCLC patients who progress during first‐line TKI treatment, the frequency of false‐negative results for p.T790M detection in ctDNA remains high (approximately 20%‐30%). This is in part due to limited levels of ctDNA and is highly dependent on the method used for mutational analysis.^18^, ^19^ Given these considerations, the objective of the present study was to assess the utility of detecting mutations in ctDNA and in mimicking circulating tumor cells (mCTCs) using the digital PCR OncoBEAM™‐*EGFR* assay compared to NGS‐56G to detect the p.T790M *EGFR* mutation in paired samples. As an exploratory analysis, we wondered whether p.T790M could be detected from mCTC using the ClearCell FX device (Biolidics Limited, Science Park, Singapore 118257).

## 2 MATERIALS AND METHODS

### 2.1 Patients

Samples were prospectively collected within the framework of the CIRCAN (“CIRculating CANcer”) project based at Lyon University Hospital. In this analysis, we used plasma samples from patients with a known *EGFR* sensitizing mutation, whose disease progressed after receiving first‐line *EGFR* TKI therapy (afatinib, erlotinib, or gefitinib, which were prescribed upon physician choice and used according to regulatory approvals) (n = 183). Plasma samples were sent to our laboratory by physicians in the context of routine detection of *EGFR* p.T790M mutation. As recommended in France, *EGFR* p.T790M substitution is screened using blood‐based samples as the first intent of tumor genotyping in this setting.^20^ Blood sampling was primarily performed when physicians documented disease progression (according to RECIST 1.1 criteria) during regular follow‐up CT‐scans (usually performed quarterly). All tumor cases were histologically or cytologically confirmed on FFPE biopsy specimens or cytological samples at initial diagnosis and *EGFR* sensitizing mutation detection was performed either on FFPE tumor samples or using cfDNA in case of tumor tissue genotyping failure as part of routine practice. For analysis of ctDNA at progression, extraction and mutation detection steps were performed by investigators who did not have access to or prior knowledge of clinical data including results from initial *EGFR* sensitizing mutation detection tests.

In this cohort, a subset of patients underwent several serial cfDNA analyses as recommended by their physician due to suspicion of progression at the time of CT‐scan. Most were patients, clinically asymptomatic, but showed evidence of radiological progression. All samples were processed similarly.

### 2.2 Sample collection

Plasma was prepared from 20‐30 mL of blood collected in K_2_ EDTA tubes (BD, 367525, 18 mg). All blood samples were delivered to the laboratory within 24 hours after collection. Detailed preanalytical considerations have previously been published.^10^

### 2.3 DNA extraction from plasma

Cell‐free DNA was extracted from 4 mL or 8 mL of plasma using the QIAamp Circulating Nucleic Acid Kit (Qiagen, Cat No 55114, Valencia, CA, USA), with a Qiagen vacuum manifold following the manufacturers’ instructions. cfDNA was then eluted in a final volume of 60‐110 µL elution buffer (AVE), depending on the volume of plasma used for the extraction (4 mL or 8 mL).^10^

### 2.4 *EGFR* p.T790M detection using BEAMing assay

cfDNA was analyzed for *EGFR* p.T790M mutation with BEAMing using the OncoBEAM™‐*EGFR* assay (Sysmex Inostics) according to the manufacturer's instructions. This particular version of the assay detects only the p.T790M and not other *EGFR* sensitizing mutations, such as p.L858R and Exon 19 deletions (DelEx19). All experiments were conducted at the Lyon Universitary Hospital. Briefly, as described previously,^11^ the cfDNA sample was diluted in 123 µL of AVE buffer (Qiagen). A PCR Master Mix, specifically targeting p.T790M was mixed with the cfDNA samples and split into six replicate of 65 µL reactions in the initial target‐specific spanning PCR. Five microliters of PCR product, diluted in low EDTA‐TE buffer (pH 8.0) was used for emulsion PCR. After breaking the emulsion PCR reaction, WT and mutant‐specific probes were hybridized and flow cytometry analysis was conducted using a cytometer (BD Bioscience, Erembodegem, Belgium). The threshold of positivity is defined by two parameters: the MAF had to exceed 0.02% and the absolute number of mutant beads had to be greater than 50.^11^ All samples with lower mutated bead counts were considered negative, even if the MAF exceeded 0.02%, this is most likely due to a low cfDNA input amount, typically below 2 ng. Polyclonal beads were detected according to their Poisson distribution.

### 2.5 Targeted next‐generation sequencing 56G oncology panel (NGS‐56G)

cfDNA libraries were created using the multiple targeted amplicon technology provided by Swift Biosciences according to the manufacturer's instructions (56G Oncology Panel Kit, Swift Biosciences, Ann Arbor, MI, Cat. No AL‐56248).^11^ The NGS‐56G assay covered the main *EGFR* exons (18, 19, 20 and exon 21) in which *EGFR* sensitizing mutations are usually detected (90% of all *EGFR* activating mutations^21^) and the p.T790M. The assay also covered mutations in 55 other genes, including *KRAS*, *MET*, and *TP53*. Fastq files, obtained by the demultiplexing of Base‐Call Files (BCL), were analyzed with the Sophia DDM Platform version 5.1.9 (Sophia Genetics, Saint‐Sulpice, Switzerland). The human genome Hg19 (GRCh37.p5) was used as the reference genome. The resulting Binary Alignment Map (BAM) files were realigned for soft‐clipping regions to recover potential indels. Variant calls were conducted by comparing the nonreference base against the averaged mean error of the corresponding averaged base quality for a given position. The subsequent Variant Call Files (VCF) were subjected to cross sample background filtering with potential artifacts removed below three standard deviations of the mean background noise for each position. Filter criteria for variant calling were set to an absolute mutated allele read counts ≥50, a minimal total depth >500 X, and a MAF threshold ≥0.5%.^11^

### 2.6 Positive and negative cell lines for the p.T790M *EGFR* Mutation

In an exploratory analysis, we assessed the performance of *EGFR* p.T790M detection in mCTC using the ClearCell FX assay (Biolidics). We first assessed the cell recovery rate of the ClearCell FX device using a cancer cell line (PC‐3) with a size greater than 15 µm. Cells were cultured in DMEM medium supplemented with 10% of Fetal Bovine Serum (Gibco, MD, USA) and 1% Penicillin/Streptomycin (Gibco, MD, USA) at 37°C with 5% of CO_2_. The microfluidic ClearCell FX system using a low‐pressure vacuum system which isolates CTCs based on size exclusion (> 11 µm) was used as previously described.^22^ PC‐3 cells (18 µm)^23^ were stained with 2 µL of CellTracker™ Green CMFDA Dye live cell stain (ThermoFisher–C2925, Waltham, USA 02451) per 10 000 cells and numbered under a fluorescent microscope. Stained cells were then spiked into healthy whole blood and a cell lysis was performed to remove red blood cells. After the enrichment run, the output tube containing enriched and stained cells was centrifuged at 500 g for 10 minutes. The supernatant was carefully removed and the pellet was resuspended in 100 µL of PBS. Immunofluorescence staining was performed using an antibody anti‐CD45 labeled with Alexa Fluor 647 (Biolegends, #304018) to differentiate mCTCs from white blood cells and assess the mCTC recovery after enrichment with the ClearCell FX. The cells were numbered with a fluorescent microscope. The number of recovered cells was compared to the input of stained cells (n = 16) and a recovery ratio was calculated.

Following CTC recovery, we investigated whether p.T790M *EGFR* mutation was detectable in mCTCs, using the p.T790M‐positive cell line NCI‐H1975 (15 µm).^24^ To examine the specificity of p.T790M detection, the p.T790M‐negative cell line HCC827 (19 µm)^25^ was used. These NCI‐H1975 and HCC827 cell lines were purchased from American Type Culture Collection (ATCC, Teddington, UK). These cells were cultured in RPMI‐1640 (ATCC) complemented with 10% of Fetal Bovine Serum (Gibco) and 1% Penicillin/Streptomycin (Gibco) and grown at 37°C with 5% CO_2_. Cells were stained with a fluorescent dye (ThermoFisher‐C2925) and were numbered with a fluorescent microscope. Then, defined numbers of fluorescent tumoral cells, or mCTCs, (5 to 100 cells/sample) were (i) stored at −20°C to evaluate the performance of p.T790M detection without white blood cells, or (ii) spiked into healthy whole blood and enriched with the microfluidic ClearCell FX (Biolidics) device according to the manufacturer’s recommendations. Finally, DNA of these isolated tumoral cells with these two conditions were extracted using the Qiagen Micro kit extraction (Qiagen, Cat No./ID: 56304, Valencia, CA, USA) and analyzed with both OncoBEAM™‐*EGFR* and NGS‐56G assays as specified above.

### 2.7 Ethical considerations

The CIRCAN study was considered to be an observational study by the local ethics committee of Lyon (Ref L15‐74; 04/29/2015). As required, the study was declared to the local authorities, since patient health data were recorded (Ref 15‐045; 05/15/2015). Furthermore, all of the patients were given detailed information about the present study and signed a written consent form. All of the samples and medical data used in the CIRCAN study were anonymized.

### 2.8 Statistical analysis

Statistics were performed using the latest version of GraphPad InStat software Version 6 (GraphPad Software, Inc, La Jolla, CA, USA). Normal distribution of continuous variables was assessed with the Shapiro‐Wilk normality test. We used the Mann‐Whitney test for nonnormally distributed variables and the Student t‐test for normalized data. Binary logistic regression was used to assess the association between occurrence of p.T790M mutation detection and the assay. All tests were 2‐sided and a *P*‐value <0.05 was considered statistically significant.

## 3 RESULTS AND DISCUSSION

### 3.1 Accuracy of p.T790M *EGFR* mutation detection assays

**Figure 1:**
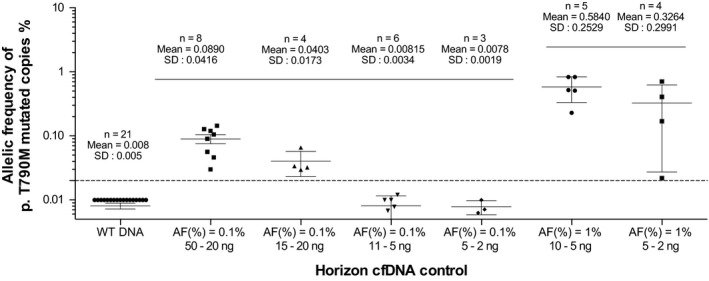
Mutated allelic frequency of p.T790M mutation observed in wild‐type control and Horizon cell‐free DNA control mutated at 0.1% and 1% using the OncoBEAM™‐epidermal growth factor receptor assay. The number of experiments is annotated and for each case, the mean and standard deviation is calculated. The error bars correspond to the standard deviation

To assess the specificity and sensitivity of the OncoBEAM™‐*EGFR* assay for p.T790M detection, WT and mutated p.T790M *EGFR* Horizon cfDNA controls were used at different allelic frequencies (0.1% and 1% MAF) (Figure 1). First, specificity of the assay was determined using the WT Horizon cfDNA control (n = 21). These experiments revealed a mean of 13 (SD ± 6) samples with mutant copies corresponding to a MAF of 0.0082% (SD ± 0.005%), well below the assay cutoff of 0.02%. Second, we assessed assay sensitivity using Horizon cfDNA control p.T790M mutated with different levels of cfDNA input (from 2 to 50 ng). The BEAMing assay was able to detect the p.T790M mutation if the cfDNA input was greater than 15 ng (n = 4) for an allelic frequency set at 0.1% MAF. To detect p.T790M at 1% MAF, an even lower cfDNA input of 2 ng was required (n = 4). The performance of the assay at these low DNA concentrations allowed us to validate a workflow for the assay to routinely perform early and/or low abundance detection of p.T790M in cfDNA at disease progression. In parallel, the sensitivity of the NGS‐56G assay was determined to be 80% and 91% for 2 and 5 ng cfDNA inputs, respectively, using the TruQ‐7 Horizon Control (1%‐1.3% p.T790M MAF) (data not shown). Analyses of assay sensitivity at level of 0.1% MAF was not studied using NGS‐56G, since the threshold of sensitivity of 0.5% MAF is the lowest limit of detection for this assay.

### 3.2 Detection of *EGFR* p.T790M mutation at disease progression

Overall, 183 samples of cfDNA were tested for *EGFR* p.T790M mutation detection in parallel to compare the performance of both the OncoBEAM™‐*EGFR* and the NGS‐56G assays. Figure S1 displays the distribution of cfDNA concentration in the cohort. Most samples (84%) had a concentration range between 1 and 5 ng/µL, limiting the cfDNA input in some reactions. The mean cfDNA concentration was 3.5 ng/µL (±6.24), which is in agreement with the DNA concentrations obtained and used in our previous work.^10^, ^11^

Using the NGS‐56G assay, *EGFR* sensitizing mutations were detected in 42.1% (77/183) of plasma samples; 28.4% (52/183) were DelEx19 and 13.6% (25/183) were p.L858R. The concordance of cfDNA and tissue sample results was 98.7% (76/77) with only one mutation missed in tissue at diagnosis. Futhermore, among the patients with *EGFR* sensitizing mutations, the associated resistant mutation p.T790M was found in 42.8% (33/77) using the OncoBEAM™‐*EGFR* assay (Mean %: 1.4261 ± 3.1315) and in 25.9% (20/77) using the NGS‐56G assay (Mean %: 6.2 ± 12.7%) (Table 1). Among the 107 samples in which no *EGFR* activating mutation was detected, 6.6% (7/106) were p.T790M positive (Mean %: 0.1146 ± 0.1102) using the OncoBEAM™‐*EGFR* assay and 0/107 were p.T790M positive using the NGS‐56G assay (Tables 1 and 2).

**Table 1:** Table summarizing the distribution of p.L858R and DelEx19 positive and negative patients found with NGS‐56G assay

Resistance Mt	Princeps Mt			
	OncoBEAM™‐*EGFR* p.T790M		NGS‐56G	
	p.L858R or DelEx19 positive	p.L858R or DelEx19 negative	p.L858R or DelEx19 positive	L858R or DelEx19 negative
p.T790M positive (N; %)	33 (43.8%)	7 (6.6%)	20 (25.9%)	0
Mean allelic frequency in % (SD)	1.4261 (3.1315)	0.1146 (0.1102)	6.2 (12.7)	—
p.T790M negative (N; %)	44 (57.2%)	99 (92.4%)	57 (73.7%)	106 (100%)
Mean allelic frequency in % (SD)	0.0084 (0.0074)	—	0.01 (6 × 10^−19^)	—
Total (N)	77	106	77	106

EGFR, epidermal growth factor receptor; NGS, next‐generation sequencing.

For these two categories, the number of patients harboring the resistance mutation p.T790M is annotated for OncoBEAM™‐*EGFR* assay and NGS‐56G assay, respectively.

**Table 2:**
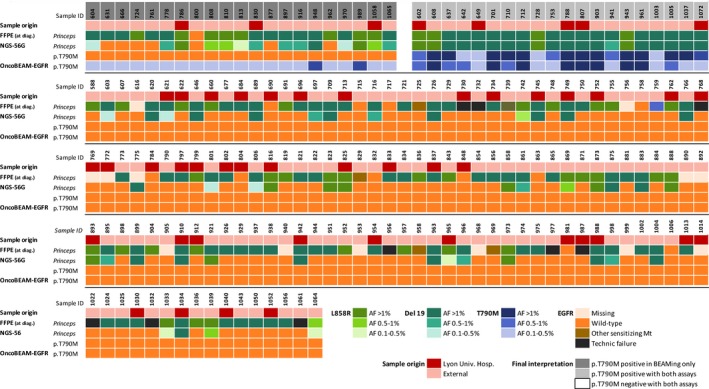
Heatmap representing the cell‐free DNA (cfDNA) sample origin, the associated FFPE results (at initial diagnosis), as well cfDNA results: (a) the principal epidermal growth factor receptor (*EGFR*) mutation detected (at disease progression) and (b) the resistance mutation (at disease progression) detected with both the OncoBEAM™‐*EGFR* and the NGS‐56G assays for the whole cohort

With respect to the overall detection of *EGFR* p.T790M mutation in all plasma samples, 21.8% (40/183) were positive for the p.T790M mutation using the OncoBEAM™‐*EGFR* assay, whereas only 10.9% (20/183) samples were positive using the NGS‐56G assay (Tables 1 and 2). The odds ratio of detecting a p.T790M mutation was 7.8‐fold higher using the OncoBEAM™‐*EGFR* assay *versus* the NGS‐56G assay (78.01 [IC95%: 4.629‐1315] for OncoBEAM™‐*EGFR* assay and 10.96 [IC95%: 4.499‐26.72] for NGS‐56G assay).

**Figure 2:**
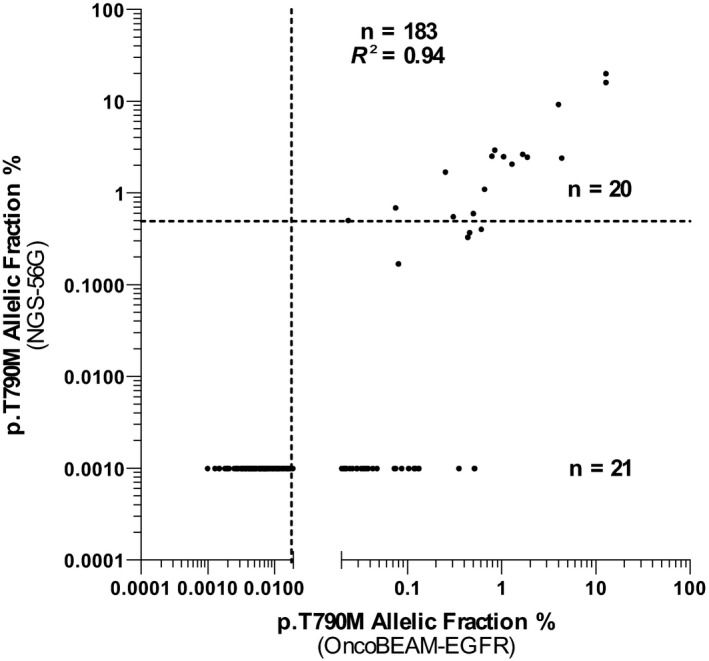
Correlation of p.T790M allelic frequency between the OncoBEAM™‐epidermal growth factor receptor (*EGFR*) assay and the NGS‐56G assay. The correlation is good with an R^2^ at 0.94. The dotted lines represent the threshold of positivity: 0.5% for NGS assay (horizontal) and 0.02% for OncoBEAM™‐*EGFR* assay (vertical). 20 concordant cases and 21 discordant cases were observed

A strong correlation was found between the OncoBEAM™‐*EGFR* assay and the NGS‐56G assay for p.T790M detection with 20 concordant cases (R^2^ = 0.94) (Figure 2). The 21 discordant results (ie, p.T790M positive using OncoBEAM™‐*EGFR*, but p.T790M negative using the NGS‐56G assay) were found to contain low *EGFR* p.T790M MAF; the MAF values detected using OncoBEAM™‐*EGFR* in these discordant samples were on average, less than 0.50% (Mean = 0.13% ± 0.14%).

Approximately 35% of the NSCLC patients harboring an *EGFR* activating mutation at diagnosis and treated with *EGFR* TKI acquired a mutation at progression, with the p.T790M point mutation comprising the largest percentage.^26^, ^27^ The p.T790M resistance mutation results in a substitution of a threonine for methionine in the *EGFR* protein which is known to increase the binding affinity of the *EGFR* to ATP, thereby activating the *EGFR* signaling pathway and leading to disease progression and drug resistance. The *EGFR* p.T790M mutation has been found copresent in therapy‐resistant NSCLC patients along with *EGFR* activating mutations (p.L858R and DelEx19) which reduced first generation *EGFR* TKI binding.^28^, ^29^, ^30^ Structural analyses via crystallography of the *EGFR* protein have demonstrated that the point mutation p.T790M leads to steric hindrance of the TKI and induces treatment resistance.^31^ There have been two main hypotheses provided to explain the occurrence of the p.T790M mutation at progression: (a) de novo acquisition of the mutation, and/or (b) preexisting p.T790M clones in low abundance/aggressiveness that are encouraged to emerge under TKI selective pressure.^27^, ^32^ De novo acquisition of the p.T790M mutation during therapy administration is the more unlikely explanation, since this has been observed to occur in less than 1% of cases.^33^, ^34^, ^35^ Therefore, the more likely mechanism of p. T790M mutation occurrence at resistance is by selection and then outgrowth of preexisting clones that contain this mutation. Zou et al 36 proposed two hypotheses explaining acquisition of p.T790M under TKI pressure: (a) residue 790 in *EGFR* is positioned at the back of the entrance of ATP binding site and steric hindrance induces the change of methionine in threonine; and (b) the substitution of methionine to threonine increases the affinity to ATP and reduces the TKI binding. They found that tumors harboring the DelEx19 activating mutation had less stability around residue 790 than tumors containing p.L858R; this in part explains why there may be a greater prevalence of NSCLC patients in which p.T790M copresent with DelEx19 vs patients who copresent with L8588R.^36^, ^37^

At least one somatic mutation according to the COSMIC database (including *EGFR* and other genes) was found in 104/183 (56.8%) of samples using the NGS assay. Among samples with no *EGFR* mutations (*EGFR* activating and/or p.T790M), we found that 15% of these patients (15/100) had *TP53* somatic alterations (MAF range 0.8%‐34%), with 32.5% of the *TP53* mutations occurring in exon 5 and another 30.2% occurring in exon 8, respectively. Other mechanisms of TKI resistance in NSCLC patients in which p. T790M mutations are not detected have also been described; this includes activation of downstream signaling pathways (mainly through *MET* or *HER2* amplifications), epithelial‐mesenchymal transition, transdifferenciation to small cell lung cancer, as well as other unknown mechanisms.^26^, ^27^, ^38^ *TP53* somatic alterations in NSCLC patients have been shown to be associated with poor outcomes.^39^ Canale et al found a similar proportion of *TP53* mutant cases and showed that *TP53* mutations in exon 8 were also associated with poor outcomes.^40^

### 3.3 Application of the OncoBEAM™‐*EGFR* assay in CTCs

**Figure 3:**
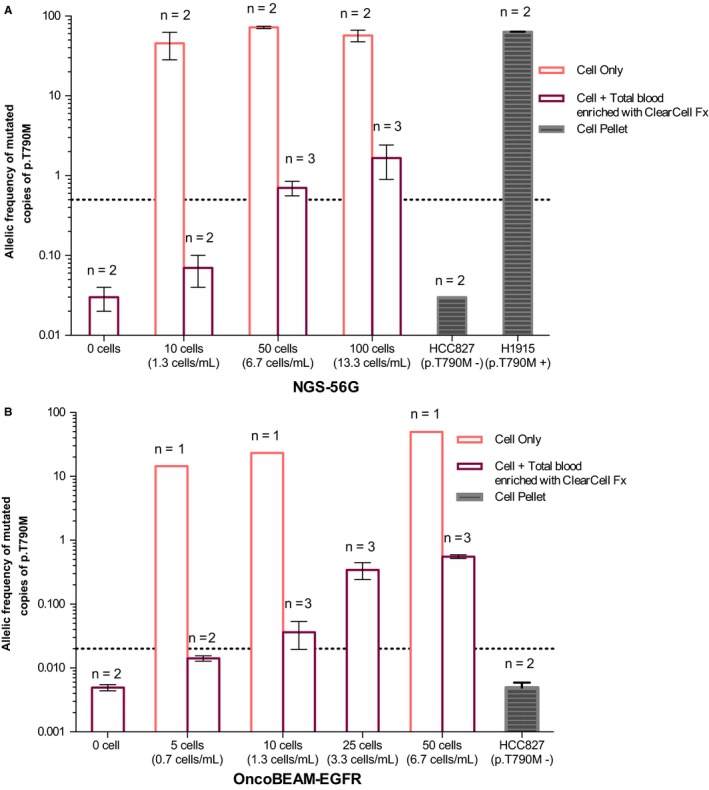
Histogram of allelic frequency of p.T790M detected using the NGS‐56G assay (A) and using the OncoBEAM™‐epidermal growth factor receptor (*EGFR*) assay (B). A defined number of cells, between 0 and 200, was either directly analyzed using NGS or BEAMing (pink bars), or first spiked into total blood, enriched using ClearCell FX, and analyzed using NGS or BEAMing (purple bars). Two negative samples were analyzed: one was total blood sample without spiked cells (condition “0 cells”) and one was the HCC827 negative cell line for p.T790M. The H1975 cell line was used as a positive control and for the spiking experiments. The dotted line corresponds to positivity threshold: 0.5% for the NGS 56G assay and 0.02% for the OncoBEAM™‐*EGFR* p.T790M assay

During the course of the present study, we set up a new microfluidic CTC platform from Biolidics, which isolates CTCs according to size and physical properties. First, we assessed the recovery rate of spiked PC‐3 tumoral cells (diameter greater than 15 µM) into 7.5 ml of total blood using this device. This rate ranges from 61% to 90% for cell input varying from 50 to 200 cells (Figure S2). Next, to evaluate the performance of NGS‐56G and OncoBEAM™‐*EGFR* assays, we used established cell lines as negative and positive controls (HCC827 and NCI‐H1975, respectively) alone and spiked into healthy total blood. The mCTCs spiked into whole blood were enriched using the ClearCell FX platform prior to mutation detection. The presence of *EGFR* p.T790M in the NCI‐H1975 positive cell line was confirmed using the NGS‐56G assay with a MAF around 65%. By contrast, the p.T790M mutation signal within the samples containing the NCI‐HCC827 negative control cell line were below the limit of detection (Figure 3). To evaluate the background quantity of false‐positive *EGFR* p.T790M mutation, total blood samples were analyzed (condition “0 cell”) (Figure 3) and the MAF of the results detected had average values of approximately 0.03%; this is well below the threshold of p.T790M mutation positivity of the NGS assay. Both NGS‐56G and OncoBEAM™‐*EGFR* were capable of detecting p.T790M in as few as five cells without spiking into total blood. These first experiments were performed in order to validate the entire worflow including counting, extraction, and analysis conditions. Subsequent experiments showed that the p.T790M mutation could be detected by NGS‐56G at a low level (ie*,* in 50 cells with the p.T790M mutation in 7.5 mL of total blood) (Figure 3A), and the BEAMing assay was capable of detecting the p.T790M mutation in preparations containing as low as 10 cells/7.5 mL (Figure 3B). In preparations containing 5 cells/7.5 mL, the *EGFR* p.T790M mutation was not detected.

CTCs are released into the bloodstream from the primary tumor or metastatic sites.^41^ A CTC population is representative of the heterogeneity and the plasticity of tumoral cells of all tumoral sites into the body. These cells may undergo an epithelial to mesenchymal transition (EMT)^42^ or acquire stem cell features to sequester themselves from their initial site of origin or to migrate into secondary metastatic sites through lymph and/or whole blood systems. Currently, CTC analyses are mainly performed to gain insights of tumor progression by transcrptional and/or epigenetic mechanisms. Since CTCs are viable and present a full representation of an individual tumor cell's molecular profile (including proteins expressed, RNA and DNA), they are promising noninvasive biomarkers.^43^ Currently, assays involving CTC counts are FDA‐approved (Food and Drug Administration) for breast cancers as a prognostic indicator using the CellSearch assay. Indeed, breast cancer patients having five or more CTCs per 7.5 mL of total blood have a shorter OS (overall survival) and PFS (progression‐free survival) than patients having fewer than five CTCs.^44^ A major technical challenge remains to isolate between 1 and 100 CTCs among normal white blood cells in a typical clinical blood sample. Nevertheless, significant progress has been made during the last ten years, and many new technologies have been developed and demonstrate feasibility when applied to both isolation and purification of these CTCs in a clinical setting.

Our results from the present study provide technical feasibility that the *EGFR* p.T790M mutation can be detected in CTCs using both the OncoBEAM™‐*EGFR* or NGS‐56G assay technologies. Soon et al have also shown that the number of CTCs might differ considerably depending on the disease stage; for instance, they found a mean number of 1.48 ± 1.71 CTCs in stage I‐III patients (in 5 mL of total blood) and a mean of 8 ± 9.95 CTCs in stage IV‐V patients using the CellSearch platform.^45^ Given the considerable body of existing knowledge clearly indicating that the numbers of CTCs capable of being isolated from a clinical blood sample is likely to be a significant limitation to bringing CTCs into routine use in clinical diagnostics, our findings justify the use of highly sensitive assays such as BEAMing to detect mutations in combinatorial analyses with CTCs (p.T790M assay; 10 tumor cells per 7.5 mL of total blood required to detect *EGFR* p.T790M following enrichment with ClearCell FX) (Figure 3B). In addition, Illie et al have shown that some “sentinel” cells may be detected in blood before detection of a lung cancer at CT‐scan, again emphasizing the need for sensitive assays 46 such as ClearCell FX. The results presented herein provide a strong methodological foundation to begin a prospective validation using patient samples.

## 4 CONCLUSIONS AND PERSPECTIVES

**Figure 4:**
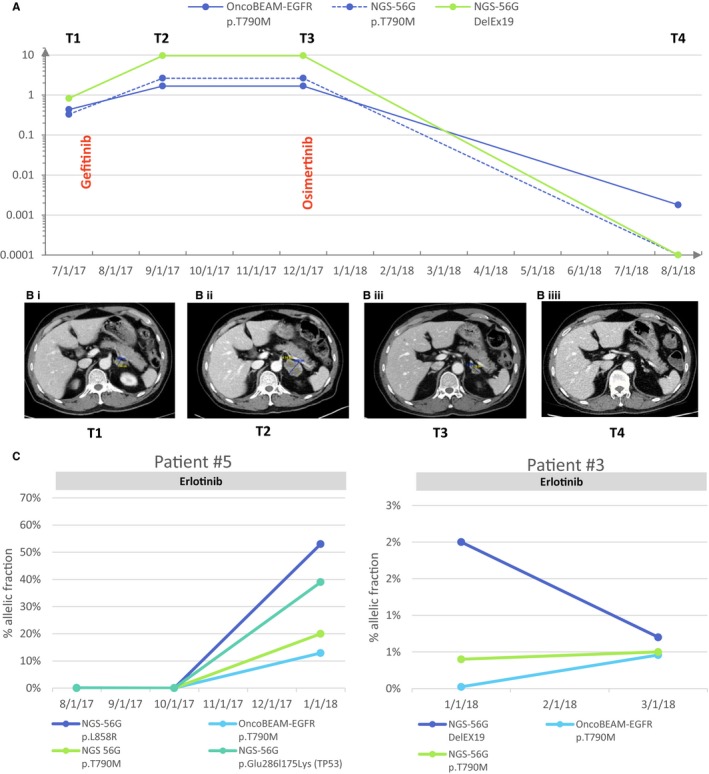
A, Monitoring of the epidermal growth factor receptor p.T790M and DelEx19 under Gefinitib treatment and Tagrisso treatment. The allelic frequency of each mutation is represented in blue for p.T790M and in red for DelEx19 for each time point available. B, Chest CT‐scan was performed at each time point. The size of primary tumor is annotated on the scan. C, Care strategy of the follow‐up of non‐small cell lung cancer patients. At diagnosis, a tissue sample was analyzed by the pathology service to define the lung cancer histology and research oncogenic drivers to personalize treatment. For each state of progression, a sampling of total blood is performed to access the cell‐free DNA, as well as to ascertain the probable mechanism of tyrosine kinase inhibitor resistance and to inform modification of treatment

The *EGFR* p.T790M mutation is the most frequent mechanism of resistance from treatment of *EGFR*‐positive NSCLC with first‐ and second‐generation TKIs. Currently, the most adopted noninvasive technique for detecting this resistance mutation at progression is cfDNA testing. In this study, we have demonstrated that the amount of DNA input is tightly associated with the accuracy and sensitivity of mutation detection with the minimal amounts of 2 ng and 15 ng that are required to detect the *EGFR* p.T790M at the level of 1% and 0.1% MAF, respectively. Since mutated copies are sometimes present at very low abundance within cfDNA, the DNA input amounts required to obtain accurate result are a major concern, and are highly assay‐dependent. Thus, high analytical sensitivity of any assay used to detect mutations at low levels of abundance in plasma samples is of paramount importance. The data from our study indicate that both the OncoBEAM™‐*EGFR* p.T790M and the NGS‐56G assays show high accuracy and mutation detection results with very good correlation (R^2^ = 0.94). However, despite this high correlation, the NGS‐56G assay failed to detect the *EGFR* p.T790M in 18/37 patients harboring the *EGFR* p.T790M at low MAF level. Nevertheless, the NGS‐56G technology offered a better coverage of *EGFR* gene mutations, where it was shown to readily detect the principal *EGFR* sensitizing mutations, as well as mutations in genes such as TP53, which have been shown to have prognostic value or involved in alternative resistance mechanisms. However, the OncoBEAM™‐*EGFR* p.T790M assay allowed us to detect the resistance mutation in 21 patients with a negative result in NGS‐56G, at lower MAF than the NGS assay could detect; this result underscores the fact that the OncoBEAM™‐*EGFR* assay has a significantly higher sensitivity for mutation detection. Tissue biopsies have demonstrated significant drawbacks (ie*,* patient discomfort, increased morbidity *via* invasive biopsy procedures, noninformative samples) for monitoring of disease and detection of targetable molecular alterations at relapse, however, a cfDNA approach can overcome these obstacles and deliver clinically actionable results. Analysis of cfDNA can be useful for noninvasive monitoring and early detection of resistance mutations such as p.T790M to assist the patient and clinician and better inform clinical decisions. To provide a more comprehensive example of the utility of using cfDNA analysis in the clinic, we fully followed the molecular and clinical evolution of a lung cancer patient during therapy using mutational analysis (Figure 4A,B). The BEAMing assay enabled us to detect a low frequency of *EGFR* p.T790M mutation that arose during therapy earlier than documented progression revealed either by symptoms or radiography. Osimertinib (third‐generation TKI) was administered as second line TKI treatment at time of radiological progression and plasma T790M detection. On the CT‐scan of December 2017, an objective decrease of the tumor (2.21 cm × 1.19 cm) was observed. The patient was still experiencing disease stabilization as of June 2018. This case report reinforces the fact that the early detection of *EGFR* p.T790M may be clinically informative and enable physicians to optimize follow‐up and timely management of NSCLC patients. Additional cases examples are reported in Figure 4C. Here, we did not assess the clinical outcomes following detection of low p.T790M MAF by OncoBEAM™‐*EGFR* assay. As a result of these initial findings, our aim is to set‐up a dedicated cohort to authorize clinical data collection to assess this critical point. Finally, to complete these initial proof‐of‐clinical concept investigations, we conducted a preliminary study to detect p.T790M in mimicking CTCs, thereby defining a threshold of mutation detection positivity for both the OncoBEAM™‐*EGFR* and NGS assay technologies to be applied to CTCs. The threshold of positivity of the OncoBEAM™‐*EGFR* p.T790M assay was five lower than NGS‐56G assay: 10 tumoral cells spiked into total blood harboring p.T790M were detected with OncoBEAM™‐*EGFR* following enrichment with ClearCell FX, while 50 cells were necessary with the 56G‐NGS assay. The fulfillment of this application has now enabled us to engage in further investigations into the timing of emergence of resistance mutations in both CTCs and cfDNA to get a complete and complementary overview of both cellular and cell‐free molecular profiles at any time in the staging and treatment plan of cancer patients with high efficiency.

## CONFLICT OF INTEREST

None declared.
