# Supplementary figures and images for: Profiling of circulating tumor DNA in plasma of non‐small cell lung cancer patients, monitoring of epidermal growth factor receptor p.T790M mutated allelic fraction using beads, emulsion, amplification, and magnetics companion assay and evaluation in future application in mimicking circulating tumor cells
Source: Cancer Med. 2019 May 21;8(8):3685–97. doi: 10.1002/cam4.2244 (PMC6866744; doi:10.1002/cam4.2244)

# Supplementary Figure 1

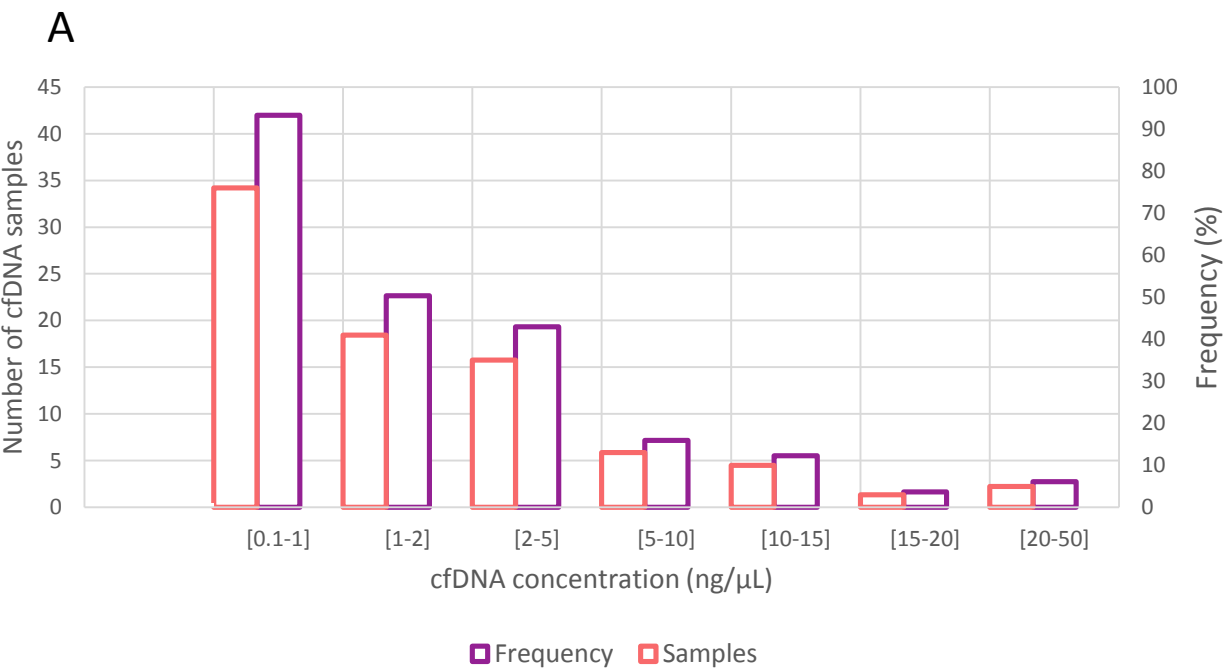

Supplementary Figure 2

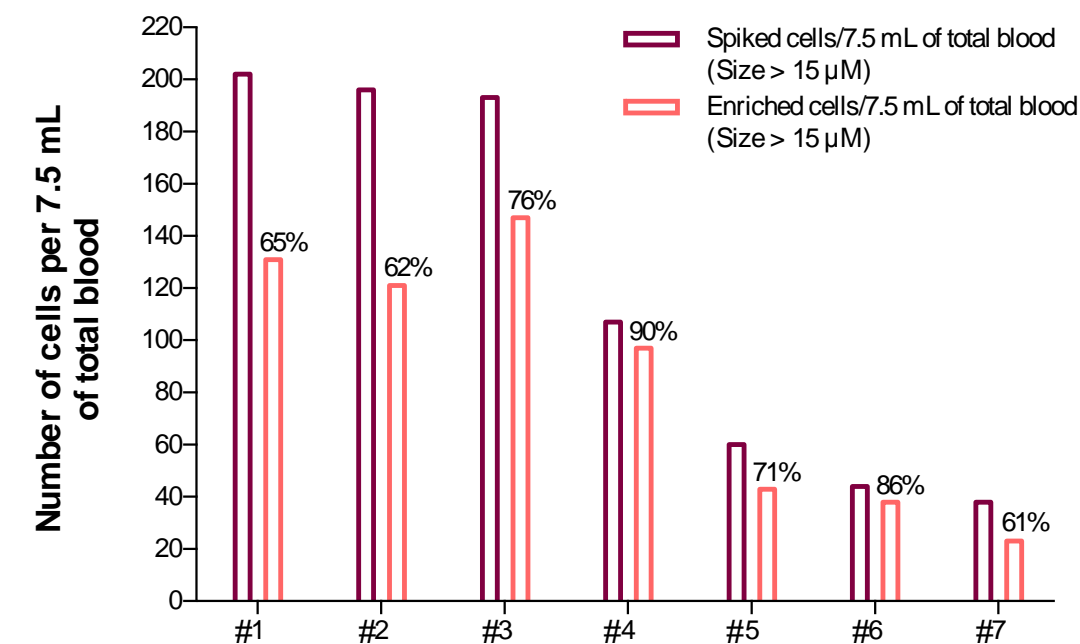

Supplement: Supplementary file 1 [file CAM4-8-3685-s001.pdf]
